# Outcomes and component-positioning in total knee arthroplasty may be comparable between supervised trained surgeons and their supervisor

**DOI:** 10.1186/s43019-019-0018-y

**Published:** 2020-01-01

**Authors:** Kazumi Goto, Yozo Katsuragawa, Yoshinari Miyamoto

**Affiliations:** Department of Orthopedic Surgery, Center Hospital of the National Center for Global Health and Medicine, Toyama 1-21-1, Shinjuku-ku, Tokyo, 162-8655 Japan

**Keywords:** Simultaneous bilateral total knee arthroplasty, Outcome, Alignment, Surgical experience

## Abstract

**Purpose:**

There are concerns that malalignment in total knee arthroplasty (TKA) occurs with less experienced surgeons. This study investigates the influence of surgical experience on TKA outcomes.

**Materials and methods:**

Nineteen patients (38 knees) who underwent bilateral TKA between 2011 and 2015 were included. A supervisor performed knee replacements associated with lower Knee Society Scores (KSS); trainee surgeons operated on the other knee. Knees were categorized into two groups: operations by the supervisor (group S) versus operations by trainee surgeons (group T). Range of motion (ROM), KSS, operative time, hip–knee–ankle angle, and femoral and tibial component angle were evaluated.

**Results:**

The mean operative time was 92.5 min in group S and 124.2 min in group T (*p* < 0.01). The mean postoperative maximal flexion was 113.2° in group S and 114.2° in group T (not significant). The mean postoperative KSS was 92.9 in group S and 93.9 in group T (not significant). No significant differences between groups in terms of proportion of inliers for the hip–knee–ankle angle, femoral component angle, or tibial component angle were observed.

**Conclusions:**

Although operative time was significantly longer for trainee surgeons versus the supervisor, no significant differences in ROM, KSS, or component positioning between supervisor and trainee surgeons were observed.

**Level of evidence:**

IV (retrospective case series design).

## Introduction

Total knee arthroplasty (TKA) is one of the most commonly performed elective procedures worldwide with excellent long-term outcomes [1, 2]. Patient survival is 99% after 1 year and 84% after 10 years [3]. Furthermore, TKA is cost-effective in the management of end-stage knee osteoarthritis [4]. However, approximately 20% of primary TKA patients are not satisfied with their outcome [5]. Outcomes of TKA result from the confluence of various factors, including hospital and surgeon procedure volume [6–8]. Several studies have suggested that surgeon experience affects the outcomes of arthroplasty [8–11]. Wilson et al. [8] demonstrated that primary TKA was a relatively low-risk surgical procedure in terms of surgical, medical, and wound complications and surgical readmissions at their institution. However, the risk of wound complications was much higher in patients operated on by junior trainees than in those operated on by more experienced surgeons. On the other hand, a study of 673 TKAs showed no difference between consultant and trainee surgeons in terms of component alignment [11].

However, it is unclear how much patient-dependent variables, for example, muscle strength, anatomical characteristics, or pain sensitivity influenced the results of these previous studies since they compared outcomes between patients. We thought it might be possible to minimize the influence of individual factors by comparing the results between the right and left knee in simultaneous TKA.

The purpose of this study was to investigate the potential differences in outcomes and component positioning between a supervisor and trainee surgeons in simultaneous TKA. The hypothesis was that the knee operated on by a less experienced surgeon would show neither poorer outcomes nor increased malpositioning of components than the knee operated on by a more experienced surgeon.

## Materials and methods

This study used a retrospective case series design (Level IV evidence), approved by the institutional review board. The files of 77 patients (154 knees) who underwent simultaneous bilateral primary TKA at our institution between December 2011 and July 2015 were reviewed. Inclusion criteria for the study were as follows: primary simultaneous bilateral TKA using the same cruciate-retaining TKA system (Vanguard® CR, Zimmer Biomet Inc., Warsaw, IN, USA) and that one knee arthroplasty was performed by the supervisor and the other arthroplasty was performed by a trainee. Exclusion criteria were as follows: follow-up less than 1 year, history of revision TKA surgery, prior osteotomies around the knee, and previous septic arthritis.

Based on these criteria, 58 patients (116 knees) had to be excluded. Complete preoperative and postoperative clinical data were available for 19 patients (38 knees). The mean patient age was 76.8 ± 5.7 years (range 64–86 years) and 17 of the patients were female. Patient characteristics are shown in detail in Table 1.

**Table 1:** Demographic data and preoperative characteristics

	Group S	Group T	*P* value
Preoperative maximal flexion (degrees)	115.3 ± 20.0	118.9 ± 16.3	0.80
Preoperative Knee Score	35.1 ± 15.0	46.1 ± 11.7	0.022
Preoperative Function Score	42.4 ± 22.3		–
Preoperative FTA (degrees)	187.8 ± 6.3	184.7 ± 3.6	0.14
Preoperative HKA (degrees)	166.2 ± 6.4	169.2 ± 3.9	0.14

*HKA* hip–knee–ankle angle, *FTA* femoral–tibial angle

All surgeries were performed under general anesthesia. A minimally invasive, mini-midvastus approach was used, and the patella was preserved. The femoral components were fixed without cement using conventional intramedullary devices. The tibial components were fixed with cement using conventional extramedullary devices. In both groups, the proximal tibia was resected first, followed by femoral resection. The aim of femoral resection was to be paralleled to the surgical epicondylar axis, with secondary referencing to the posterior condylar axis and the Whiteside line [12]. The rotation of the tibial component was determined by combining Akagi’s line [13] with the self-adjusting technique [14]. The goal of these procedures was to restore a neutral hip–knee–ankle angle (HKA) with a neutral femoral component and the tibial component angles in the coronal plane. The first replacement was performed on the knee that had a lower Knee Score (KS) on the Knee Society Score (KSS) by a supervisor who had experience of over 1000 TKAs. The same supervisor performed the procedure in all 19 patients. The other knee was then operated on by trainee surgeons who had performed fewer than 20 TKAs. The supervisor was present during all trainee procedures.

**Fig. 1:**
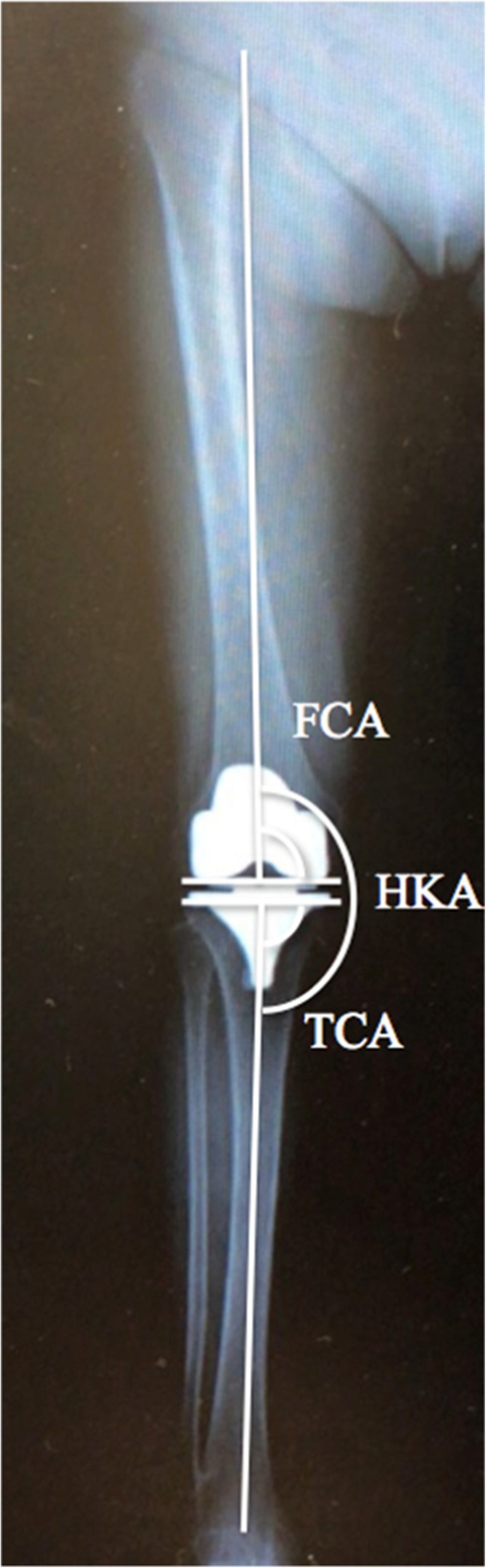
Radiograph of the postoperative component alignment in the coronal plane. HKA was measured as the *white line*. FTA was measured as the angle between the femoral shaft axis and tibial shaft axis. *FCA* femoral coronal angle, *FTA* femoral–tibial angle, *HKA* hip–knee–ankle angle, *TCA* tibial coronal angle

The knees were consecutively divided into two groups: group S with knees operated on by the supervisor and group T with knees operated on by a trainee surgeon. Clinical and radiographic assessments were performed preoperatively and at 6 weeks, 3 months, and 1 year postoperatively. The osteoarthritis (OA) grade (Kellgren-Lawrence classification) of knees was defined on preoperative X-ray films. Clinical outcome measures included range of motion (ROM) and KSS. Operative time was recorded in minutes from skin incision to wound closure. The femoral-tibial angle (FTA), HKA, and the orientation of the two components were determined based on long-leg weight-bearing radiographs 6 weeks postoperatively. The coronal alignment of the entire lower limb was assessed using the HKA. The measured angle between the coronal femoral mechanical axis and the femoral component was defined as the femoral component angle (FCA). The measured angle between the coronal tibial mechanical axis and a line through the proximal aspect of the tibial component was defined as the tibial component angle (TCA; Fig. 1). For HKA and individual component positioning, both 2° and 3° cutoff values were used for the evaluation of inliers. The varus angle was considered as a positive value, and the valgus angle was considered as a negative value. Radiographic digital measurements were performed twice at two different points in time by a single author (KG). This author was blinded as to who had operated on the examined knee. The intra-observer error was less than 2° for any measurement. The occurrence of severe complications (wound dehiscence, septic arthritis, and periprosthetic fracture) during the post-operative period was also recorded.

Preoperative demographic characteristics, operative time, ROM, and KSS were analyzed using a non-parametric test (Mann–Whitney U). Fisher’s exact test was used to assess differences in the number of inliers of component positioning and alignment between subgroups. A *p* value < 0.01 was considered statistically significant. All statistical analyses were performed using the free R software (version 3.2.1; R Development Core Team).

## Results

There were no severe postoperative complications (wound dehiscence, septic arthritis, or periprosthetic fracture) during the follow-up. The preoperative OA grade was not significantly different between the groups (*p* = 0.78). The mean preoperative KSS was 36.1 ± 15.0 in group S and 46.1 ± 11.7 in group T (*p* < 0.01). The preoperative KSS was significantly lower in group S than in group T (Table 1). The mean preoperative maximal extension was − 10.3 ± 5.5° in group S and − 7.9 ± 4.1° in group T. The mean preoperative ROM, FTA, and HKA were not significantly different between the two groups (Table 1). The mean operative time was 92.5 ± 13.6 min in group S and 124.2 ± 12.2 min in group T, which was a statistically significant difference (Table 2).

**Table 2:** Postoperative characteristics

	Group S	Group T	*P* value
Operative time (min)	92.5 ± 13.6	124.2 ± 12.2	0.00000037
Postoperative maximal flexion	113.2 ± 15.7	114.2 ± 14.3	0.84
Postoperative Knee Score	92.9 ± 9.4	93.9 ± 5.8	0.70
Postoperative function score	69.5 ± 10.6		–

The mean postoperative maximal extension was − 1.8 ± 4.9° in group S and − 1.3 ± 4.5° in group T. The mean postoperative maximal flexion was 113.2 ± 15.7° in group S and 114.2 ± 14.2° in group T (Table 2). The mean postoperative KSS was 92.9 ± 9.4 in group S and 93.9 ± 5.8 in group T. The overall mean function score (FS) of the KSS improved from 42.4 ± 22.3 to 69.5 ± 10.6 postoperatively.

The postoperative component alignment in the coronal plane in both groups is shown in Table 3. The mean postoperative FTA was 176.1 ± 2.8° in group S and 173.0 ± 3.0° in group T (*p* < 0.01). The mean postoperative HKA was 178.0 ± 2.8° in group S and 180.7 ± 2.7° in group T (*p* < 0.01). The alignment of the knee was more likely to be varus in group S than in group T (Table 3).

**Table 3:** Overall and individual component alignment in the coronal planes

	FTA	HKA	FCA	TCA
Group S	176.1 ± 2.8	178.0 ± 2.8	91.6 ± 5.2	89.1 ± 2.1
Group T	173.0 ± 3.0	180.7 ± 2.7	90.4 ± 1.9	91.2 ± 1.4
*P* value	0.0026	0.0047	0.092	0.0022

*FTA* femoral–tibial angle, *HKA* hip–knee–ankle angle, *FCA* femoral coronal angle, *TCA* tibial coronal angle

In the coronal plane, there was no significant difference between the two groups regarding the proportion of inliers at 2° or 3° cutoff values for HKA, FCA, and TCA (Table 4).

**Table 4:** The percentage of inliers in the coronal planes

	HKA	FCA	TCA
	Percentage inliers within 3° (within 2°)		
Group S	60.0 (35.0)	85.0 (75.0)	90.0 (75.0)
Group T	90.0 (75.0)	85 (75.0)	100.0 (75.0)
*P* value	0.65 (0.11)	1.00 (1.00)	0.49 (1.00)

*HKA* hip–knee–ankle angle, *FCA* femoral coronal angle, *TCA* tibial coronal angle

## Discussion

The most important finding of this study is that operative time was significantly longer in the trainee surgeon group; however, there were no significant differences in ROM, KSS, or component positioning between the supervisor and trainee surgeons. An unexpected finding was that the postoperative HKA was closer to neutral with trainee surgeons, which had been set as a goal for these surgeries, than the experienced surgeon.

Several studies have demonstrated that higher surgical and hospital volumes are associated with more favorable patient outcomes [8–10]. Patients operated on by low-volume surgeons were more likely to report an inability to flex the knee to 90° and to achieve full extension at the 2-year follow-up [12]. Highly skilled surgeons achieve good outcomes and, as they gain experience, they become better in selecting patients more suitable for surgery [10]. Training and education in the treatment of a specific condition should therefore lead to improved outcomes [15]. However, it seems that TKA mid-term survival does not depend on surgeon volume [16]. Moreover, no association between surgeon volume and 1- and 3-year revision rates has been observed [17, 18]. It appears that the association between surgeon experience and clinical outcomes remains contentious.

Wilson et al. [8] also compared the surgical outcomes of 2272 total hip arthroplasties and 2646 TKAs performed by trainees and consultants. Their research demonstrated that there were no associations between complications, transfusion rate, or surgical readmissions. Furthermore, they found that whether a consultant or trainee had performed the procedure had no significant effect on outcomes. Other similar studies have reported that outcomes may be comparable between trainees and supervisors when operations are performed under supervision or using a navigation system [19–22]. The major difference in our study was that we compared the outcomes of more and less experienced surgeons in the same patients without the use of a navigation system. The advantage of this approach is that patient-dependent variables (for example, pain sensitivity, muscle strength, bone quality) are eliminated. Traditional parallel group studies may have a potential for bias. There were no significant differences in the preoperative KSS between the two groups, although it is difficult to ignore the influence of this gap in preoperative knee conditions.

Several studies have reported that accurate component positioning improves functional outcomes [23–25]. Huang et al. [26] reported that accurate coronal alignment of total knee prostheses (to within 3° of neutral) resulted in better function and better quality of life up to 5 years postoperatively. A study by Mahaluxmivala et al. [11] compared component positioning between consultant and trainee surgeons in 674 TKAs. They found that there was a trend toward more accurate component positioning by consultants. However, the alignment was not significantly different. In our study, the rates of outliers were not significantly different between the two groups, but the HKA was closer to neutral in the trainee group. One reason for this might be that operative time in the trainee group was significantly longer, possibly for component positioning and alignment evaluation under the direct supervision of the supervisor. Consequently, there is a possibility that results might be different if surgery was performed by the trainee alone without such supervision.

This study has several limitations that should be acknowledged. First, the study had a selection bias. Trainee surgeons were more likely to operate on easier cases whereas the supervisor operated on the more severely degenerated cases. In fact, the knees operated on by the supervisor had slightly lower preoperative KSS. Therefore, this may have the effect of reducing the difference in outcomes between the two groups. In addition, it is possible that, if a patient is not satisfied with one knee, it will affect the self-reported outcome of the other knee as well. Second, only one supervisor performed all the operations in this study. There is, therefore, a possibility that the surgical technique of this supervisor was suboptimal. However, the mean postoperative KSS was over 90 in both groups. Third, all trainee surgeries were performed under the direct supervision of this supervisor. Hence, this study was not a comparison of surgical outcomes between a supervisor and trainees when operating independently. Fourth, the long-term results in our study are unknown, and a longer follow-up is required to confirm findings. Finally, the number of patients was very small, and the results might vary from those of studies with larger sample sizes, especially for the comparison of complications. This is the biggest limitation of this study; thus, further large-scale studies are required for better comparisons.

Despite these limitations, however, no prior study in the orthopedic literature has compared the outcomes between a supervisor and trainee surgeons in simultaneous TKA. Our results are a good reference for educational institutions as it eliminated patient-related individual factors influencing outcomes by allowing one patient to serve as the experiment and the control.

## Conclusions

Although operative time was significantly longer for trainee surgeons versus the supervisor, no significant differences in ROM, KSS, or component-positioning were observed between the supervisor and trainee surgeons.

## Data Availability

The datasets from and/or analyzed during the current study are available from the corresponding author on reasonable request.

## Abbreviations

FS: Function score
FTA: Femoral–tibial angle
HKA: Hip–knee–ankle angle
KS: Knee Score
ROM: Range of motion
TKA: Total knee arthroplasty
